# Sexual behaviors, contraception use and barriers among adolescents and young adults in rural Haiti

**DOI:** 10.1186/s12905-023-02268-5

**Published:** 2023-03-27

**Authors:** Abbey R. Masonbrink, Emily A. Hurley, Nikolaus Schuetz, Jonathan Rodean, Emily Rupe, Kemi Lewis, Marie Daphnée Boncoeur, Melissa K. Miller

**Affiliations:** 1 Children’s Mercy Hospital, Kansas City, MO US; 2 Department of Pediatrics, University of Missouri-Kansas City School of Medicine, Kansas City, MO US; 3 Children’s Hospital Association, Lenexa, KS USA; 4 Division of Health Services and Outcomes Research, Children’s Mercy, Kansas City, MO US; 5 Department of Population Health, University of Kansas Medical Center, Kansas City, MO US; 6 University of Kansas School of Medicine, Wichita, KS US; 7 Global Birthing Home Foundation, Leawood, KS US; 8 Department of Pediatrics, Children’s Mercy Kansas City, 2401 Gilham Rd, Kansas City, MO 64108 US

**Keywords:** Contraception, Pregnancy prevention, Adolescents and young adults, Haiti

## Abstract

**Background:**

Adolescents and young adults (AYAs) in Haiti experience a high unintended pregnancy rate, in part due to unmet contraception needs. Little is known about AYA opinions of and experiences with contraception that may explain remaining gaps in coverage. We aimed to describe barriers and facilitators to contraception use among AYAs in Haiti.

**Methods:**

We conducted a cross-sectional survey and semi-structured qualitative interviews with a convenience sample of AYA females aged 14–24 in two rural communities in Haiti. The survey and semi-structured interviews assessed demographics, sexual health and pregnancy prevention behaviors and explored contraception opinions and experiences according to Theory of Planned Behavior constructs: attitudes, subjective norms, and perceived behavioral control. We used descriptive statistics to report means and responses to Likert scale and multiple-choice questions. Guided by content analysis, we analyzed interview transcripts through inductive coding and team debriefing.

**Results:**

Among 200 survey respondents, 94% reported any past vaginal sexual activity, and 43% reported ever being pregnant. A large majority were trying to avoid pregnancy (75%). At last sexual activity, 127 (64%) reported use of any contraceptive method; Among them, condoms were the most common method (80%). Among those with previous condom use, most reported use less than half the time (55%). AYAs were concerned about parental approval of birth control use (42%) and that their friends might think they are looking for sex (29%). About one-third felt uncomfortable going to a clinic to ask for birth control. In interviews, AYAs desired pregnancy prevention but frequently noted concerns about privacy and parental, community and healthcare provider judgement for seeking care for reproductive health needs. AYAs also noted a lack of contraception knowledge, evident by frequent misconceptions and associated fears.

**Conclusion:**

Among AYAs in rural Haiti, a large majority were sexually active and desire pregnancy avoidance, but few were using effective contraception due to numerous concerns, including privacy and fear of judgement. Future efforts should address these identified concerns to prevent unintended pregnancy and improve maternal and reproductive health outcomes in this population.

**Supplementary Information:**

The online version contains supplementary material available at 10.1186/s12905-023-02268-5.

## Introduction

Haiti has the highest maternal and infant mortality rates in the Western hemisphere, with 480 deaths per 100,000 and 50 deaths per 1,000 live births respectively [1–3]. Hence, increasing access to pregnancy prevention strategies remains an important goal to improve health outcomes among Haitian women and children. The United Nations Sustainable Development Goals and Family Planning 2020 were created to ensure universal access to sexual and reproductive health (SRH) care for women living in the low and middle income countries (LMICs) [3]. Haitian women, especially younger women and adolescents, are among those with the least access to effective hormonal contraception methods, (e.g., pills, intravaginal ring, injectable, long acting reversible contraception [LARC]) compared to others living in LMICs in the Western Hemisphere due to numerous barriers (e.g., cost, transportation, privacy concerns) [4, 5]. While public health clinics in Haiti are required to provide free contraception services, barriers remain, including transportation, supply issues, as well as cost if seeking care at private facilities, as well as concern about parental approval for adolescents [1]. Among Haitian women of reproductive age, less than a quarter report use of contraception and 52% have an unmet need for effective contraception, with greater unmet needs among those living in rural compared to urban communities [5]. These unmet contraception needs contributed to an estimated 413,000 unintended pregnancies among women living in Haiti in 2019 [6].

Adolescent females (aged 15–19 years) in Haiti have a pregnancy rate of 51 per 1,000, much higher than other LMICs in the Western Hemisphere; despite decreasing rates in the past two decades, 14% of Haitian adolescent females are estimated to be pregnant or have been pregnant in 2020 [1, 2]. Haitian youth report low condom use rates and experience some of the highest rates of sexually transmitted infections (STIs) in the Western Hemisphere. About 80% of new HIV infections in Haiti occur in young women [6, 7]. Among adolescents living in Port-au-Prince, 60% reported past sexual activity and 48% reported engaging in sexual behaviors (e.g., unprotected sex, multiple partners) linked with increased risk for adverse SRH outcomes [6]. Most studies of youth in Haiti have focused primarily on understanding sexual health behaviors and HIV risk. A 2003 cross-sectional school-based survey regarding HIV prevention behaviors revealed that only 18% of youth reported always/sometimes use of condoms and 43% reported three or more lifetime sexual partners [8]. Factors associated with sexual behaviors linked with adverse SRH outcomes (e.g., STIs) among adolescents in Haiti include contextual factors, including political unrest, violence and economic conditions and family characteristics, such as absence of parents, lower education and low socioeconomic status. Additionally, psychosocial and individual factors, such as impulsivity, social stigma, privacy concerns and low self-efficacy impact adolescent sexual behaviors, including birth control use, and related health outcomes [6, 8–12]. Altough sexual health services are offered at public health facilities in Haiti at no cost, adolescents still face structural barriers to accessing this care due to transportation, privacy concerns and healthcare provider concerns about parental consent, as well as cost if seeking care at private health facilities [10–12]. While numerous factors linked with increased risk of STIs have been identified in Haitian youth, no studies to date have investigated pregnancy prevention behaviors, such as attitudes and barriers regarding contraception use in Haitian adolesencents and young adults (AYAs).

The Theory of Planned Behavior (TPB) is a theoretical framework that posits behavioral intention is driven by attitudes (beliefs and values), subjective norms (perceived social expectations), and perceived behavior control [13, 14]. The TPB is a comprehensive model that has been used for understanding numerous health behaviors driven by behavioral intention. The TPB framework has been used extensively to understand sexual health behaviors and contraception use specifically in the AYA population [14, 15].

Given the high rates of STIs, unplanned pregnancy, and maternal mortality, coupled with low rates of contraception use, further work is needed to understand facilitators and barriers to contraception use among Haitian AYAs. The goal of this mixed-methods study was to use the TPB framework to describe the attitudes, subjective norms and perceived behavioral control related to contraception use among AYAs in rural Haiti.

## Methods

### Study design, setting and participants

We conducted a cross-sectional survey of female AYAs in rural Haiti to assess sexual health behaviors, barriers and facilitators to contraception use from August 2021 to March 2022. We also conducted semi-structured interviews with a subset of participants. The study was conducted in two rural communities in the Southern and Central Departments of Haiti, where our two partner organizations (Global Birthing Home Foundation, Hospital Albert Schweitzer) provide free maternal health services, including some contraception methods and condoms. Participant inclusion criteria included biological females aged 15 to 24 years old in general good health, as assessed by the research team (e.g., without severe psychiatric illness or developmental delay) who lived in one of two rural communities.

The study was reviewed (including study protocol, all study documents and verbal consent process) and approved by the institutional review board at the affiliated academic institution, Children’s Mercy Hospital, Kansas City, MO, in the United States and by our local partner organization (Global Birthing Home Foundation and Hospital Albert Schweitzer) according to local procedures in Haiti. All study materials were also reviewed and approved by a community representative at both sites in Haiti to ensure the study was ethically and culturally appropriate. All study materials were created in English and translated into Haitian Creole by a bilingual university-level interpreter (Boncoeur). The survey and interview guide were reviewed with three AYAs representative of the sample and revised based on feedback. Translated documents were reviewed by the local research team and the university faculty representative in Haiti to ensure accuracy and comprehension were retained and modifications were made based on feedback. All study procedures and data collection were conducted in Haitian Creole.

### Procedures

We worked with a local resident to identify homes in the community where potential eligibile AYAs participants may reside who were then approached at their homes and underwent verbal assent (15–17 years old) or consent (18–24 years old) for study enrollment. As our study topic may be considered sensitive and thus taboo to discuss with men or older generations, surveys and interviews were conducted by young Haitian females to maximize participant comfort. To ensure privacy during data collection, if a parent or guardian was present they were given brief study information and then asked to provide a private space for all study procedures. We obtained verbal consent to protect participant confidentiality and minimize collection of any personal identification information. The survey was administered verbally by the study team and data were entered and managed using Research Electronic Data Capture (REDCap). Upon survey completion, a convenience sample of 25 AYAs were then invited to complete a semi-structured interview which was audio-recorded. The survey took approximately thirty minutes to complete and interviews took approximately one hour. All participants were given a menstrual hygiene kit and verbal education on kit use. No participant identifiers were collected.

### Survey and semi-structured interview instruments

The survey was developed using previously validated instruments (more details below) to assess demographics, sexual health behaviors, contraception use as well as previously proven TPB constructs including attitudes, subjective norms and perceived behavioral control for contraception use [16–19]. Qualitative methods (i.e., interviews) were used to supplement our quantitative findings, specifically to elicit rich individual perspectives on pregnancy prevention attitudes and experiences. The guides were based on the TPB constructs and included in the appendix.

*Demographics and Sexual Behaviors* We assessed socio-demographics with questions adapted from a previous survey that examined SRH among adolescents in Haiti [20]. These multiple choice questions assessed age, marital status, school attendance, religion and home environment (e.g., “What best describes where you live?, Options: In a house; Under a tent; Other”) [6]. We assessed past sexual history and contraception use by asking yes/no questions (e.g., “Have you ever had vaginal intercourse or sex (meaning ‘penis in vagina sex’)?”) and multiple choice questions (e.g., The last time you had vaginal intercourse, which method (s) did you or your partner use to prevent pregnancy?)

*Attitudes* We used 4-point Likert scale items to assess pregnancy and contraception attitudes and intentions (e.g., “I think it would be good for me to get pregnant at this time in my life.” Options: Very true; Sort of true; Not very true; Not at all true; “In general, birth control is too much of a hassle to use.” Options: strongly agree, agree, disagree, strongly disagree). The interview guide included open-ended questions to explore pregnancy and contraception attitudes (e.g., Thinking about young women in your community, what would you say are their biggest worries when it comes to their sexual health?; What kinds of things in your life are important to you when making a decision about pregnancy?).

#### Subjective norms

We assessed subjective norms about contraception use by asking 4-point Likert scale survey items (e.g., It would be hard to get a boy to use birth control with me; My parents wouldn’t approve of me using birth control; If I used birth control, my friends might think that I was looking for sex; Options: strongly agree, agree, disagree, strongly disagree). For the interview guide we assessed subjective norms by asking open-ended interview questions (e.g., If you were to get pregnant right now, how do you think your partner(s) would react?).

#### Perceived behavioral control

We assessed perceived behavioral control by asking 5-point Likert scale survey items (e.g., “I don’t feel comfortable going to a clinic to ask for birth control. Options: strongly disagree, disagree, agree, strongly agree”). We also assessed perceived behavioral control by asking open-ended interview questions (e.g.,. How do you think it would make you feel to be offered counseling and birth control at a clinic?).

### Data analysis

For quantitative analysis, we used descriptive statistics to report means and standard deviation for normally distributed continuous data. Categorical data were presented as proportions. There were no missing data. Because pregnancy intentions and contraception experience differ by developmental stage we present the data from all participants and also within three age categories (i.e., 14–17, 18–21, and 22–24 years old) [21–23]. Descriptive statistical analyses were conducted using SPSS, Version 20 or SAS software v 9.4 (SAS Institute, Cary, NC, USA).

For qualitative analysis, interview audio-recordings were transcribed and translated from Haitian Creole to English and uploaded into Dedoose (Version 4.12). We used an iterative process to identify emergent themes within and across interviews. A coding tree was developed based on the interview guides and was periodically revised to include relevant inductive codes as emergent themes arose. The first three interviews were coded together by three members of the study team (Masonbrink, Hurley, Schuetz) to develop mutually agreed upon definitions for each code and to establish examples of each code; codes were reviewed and revised, and the interviews were again coded by the same 3 members of the study team. Any disagreements in coding were resolved by consensus. Each interview was then coded separately by 1–2 study team members. After coding, the three team members met to discuss the results; again, any disagreements in coding were resolved by consensus. Memos of coding decisions were kept to provide consistency in coding as the coding progressed. The coding team summarized coding outputs, synthesizing major themes according to TPB constructs.

## Results

### Quantitative findings

#### Participant demographics

Among 200 survey respondents at the two study sites, most were 18 to 21 (n = 76, 38%) or 22 to 24 (n = 71, 36%) years old and approximately half (n = 102, 51%) reported regular school attendance (Table 1). Of the 200 participants, 25 also completed qualitative interviews.

**Table 1:** AYA Participant Characteristics

	N = 200, N (%)
Age Category (years)	
14–17	53 (27)
18–21	76 (38)
22–24	71 (36)
Highest education level (grade)	
1-6th primary school	35 (18)
7-9th primary school	70 (35)
3-4th secondary school	83 (42)
Graduated secondary school	12 (6)
Marital status	
Yes	7 (4)
Regular school attendance (3 or more days per week)	
Yes	102 (51)
Living situation	
House	178 (89)
Tent	22 (11)
Other	0 (0)
Religion	
Catholic	63 (32)
Other Christian religion	96 (48)
Other	1 (1)
None	39 (20)
Did not answer	1 (1)
Sexual Orientation	
Gay	0 (0)
Lesbian	0 (0)
Straight/not gay or lesbian	199 (99)
Bisexual	1 (1)
Other	0 (0)
Did not seek care in past year	
Yes	100 (50)

#### Sexual behaviors and contraception use

One hundred eighty-seven (94%) reported any past vaginal intercourse (Table 2). Eighty-six (43%) were 14–17 years old at first sexual intercourse and 136 (68%, missing data = 29) reported two or fewer lifetime partners. Nearly half (n = 85, 43%) reported ever being pregnant; of these, 13 had a previous abortion. Most (n = 127, 64%) reported use of any contraceptive method at last sexual activity (Table 3). At last sexual activity, approximately half (n = 101, 51%) reported condom use and some (n = 25, 13%) reported medroxyprogesterone acetate use. When asked about frequency of condom use, most reported “never use” (n = 42, 21%) or use “less than half the time” (n = 109, 55%).

**Table 2:** AYA Sexual Behaviors and Pregnancy Intentions

	Total N = 200	Age 14-17 N = 53	Age 18-21 N = 76	Age 22-24 N = 71
Any sexual activity				
Yes	187 (94)	44 (83)	74 (97)	69 (97)
Oral sexual activity	52 (26)	11 (21)	25 (33)	16 (23)
Vaginal sexual activity	187 (94)	44 (83)	74 (97)	69 (97)
Anal sexual activity				
Age at first sexual activity (years)				
13 years or younger	16 (8)	8 (15)	5 (7)	3 (4)
14–16	86 (43)	26 (49)	34 (45)	26 (37)
17 or older	61 (31)	4 (8)	25 (33)	32 (45)
Missing	37 (19)	15 (28)	12 (16)	10 (14)
Number of lifetime partners				
0	8 (4)	6 (11)	1 (1)	1 (1)
1	64 (32)	18 (34)	22 (29)	24 (34)
2	64 (32)	15 (28)	27 (36)	22 (31)
3	22 (11)	3 (6)	9 (12)	10 (14)
4 or more	13 (7)	2 (4)	6 (8)	5 (7)
Missing	29 (15)	9 (17)	11 (15)	9 (13)
Ever been pregnant				
Yes	85 (43)	9 (17)	40 (53)	36 (51)
No	110 (55)	40 (76)	35 (46)	35 (49)
Did not answer	14 (17)	1 (11)	5 (13)	8 (22)
If Yes to ever been pregnant, have you ever had an abortion?				
Yes	13 (15)	2 (22)	4 (10)	7 (19)
No	58 (68)	6 (67)	31 (78)	21 (58)
Did not answer	14 (17)	1 (11)	5 (13)	8 (22)
Pregnant now				
Yes	14 (7)	1 (2)	7 (9)	6 (9)
No	178 (89)	47 (89)	67 (88)	64 (90)
Don’t know	2 (1)	0 (0)	1 (1)	1 (1)
Did not answer	6 (3)	5 (9)	1 (1)	0 (0)
Thoughts on Pregnancy				
Trying to get pregnant	2 (1)	0 (0)	1 (1)	1 (1)
Wouldn’t mind getting pregnant	13 (7)	0 (0)	3 (4)	10 (14)
Wouldn’t mind avoiding pregnancy	27 (14)	7 (13)	10 (13)	10 (14)
Trying to avoid pregnancy	149 (75)	41 (77)	60 (79)	48 (68)
Don’t know	3 (2)	0 (0)	1 (1)	2 (3)
Did not answer	6 (3)	5 (9)	1 (1)	0 (0)

**Table 3:** AYA Contraception Use

	Total N = 200, n (%)	Age 14-17 N = 53, n (%)	Age 18-21 N = 76, n (%)	Age 22-24 N = 71, n (%)
Any contraception use at last sexual activity (including condoms)				
Yes	127 (64)	31 (59)	47 (62)	49 (69)
No	37 (19)	8 (15)	17 (22)	12 (17)
Unknown	36 (18)	14 (26)	12 (16)	10 (14)
What method did you use at last sexual activity?				
None	37 (19)	8 (15)	17 (22)	12 (17)
Condom	101 (51)	29 (55)	37 (49)	35 (49)
Birth control pill	1 (1)	0 (0)	0 (0)	1 (1)
Birth control patch	0 (0)	0 (0)	0 (0)	0 (0)
Nuva Ring (or any birth control ring)	2 (1)	0 (0)	0 (0)	2 (3)
Medroxyprogesterone acetate (birth control shot)	25 (13)	5 (9)	9 (12)	11 (16)
Nexplanon or Implanon (or any implant)	3 (2)	0 (0)	2 (3)	1 (1)
Intrauterine device (IUD)	0 (0)	0 (0)	0 (0)	0 (0)
Withdrawal	3 (2)	0 (0)	1 (1)	2 (3)
Emergency contraception	0 (0)	0 (0)	0 (0)	0 (0)
Spermicide	0 (0)	0 (0)	0 (0)	0 (0)
Cervical cap	0 (0)	0 (0)	0 (0)	0 (0)
Diaphragm	0 (0)	0 (0)	0 (0)	0 (0)
When you have vaginal intercourse, how often do you use a condom?				
Never	42 (21)	8 (15.1)	17 (22.4)	17 (24)
Less than half the time	109 (55)	28 (52.8)	44 (57.9)	37 (52)
More than half the time	0 (0)	0 (0.0)	0 (0.0)	0 (0)
Always	0 (0)	0 (0.0)	0 (0.0)	0 (0)
Prefer not to answer	2 (1)	0 (0.0)	0 (0.0)	2 (3)
Missing	47 (24)	17 (32.1)	15 (19.7)	15 (21)

### Attitudes

Across all age categories a large majority were trying to avoid pregnancy (n = 149, 75%; Table 2). The most frequently identified barriers about contraception use were concerns that contraception was “too much of a hassle” (n = 57, 29%; Table 4) and “is morally wrong” (n = 37, 19%).

**Table 4:** Contraception Attitudes, Subjective Norms and Perceived Behavioral Control

Which of the following do you strongly agree/agree are a problem or barrier for you to use or get birth control?	Total N = 200, n (%)	Age 14-17 N = 53, n (%)	Age 18-21 N = 76, n (%)	Age 22-24 N = 71, n (%)
**Attitudes**				
Too much of a hassle	57 (29)	17 (32)	20 (26)	20 (28)
Is morally wrong	37 (19)	11 (21)	11 (15)	15 (21)
Interferes with sexual enjoyment	14 (7)	5 (9)	5 (7)	4 (6)
Takes too much planning	5 (3)	2 (4)	0 (0)	3 (4)
I do not have enough money to pay for birth control	5 (3)	1 (2)	1 (1)	3 (4)
Too expensive	4 (2)	1 (2)	1 (1)	2 (3)
**Subjective Norms**				
My parents wouldn’t approve of me using birth control	83 (42)	29 (55)	25 (33)	29 (41)
My friends might think I am looking for sex	57 (29)	17 (32)	22 (29)	18 (25)
Would be hard to convince male partner to use	56 (28)	18 (34)	19 (25)	19 (27)
**Perceived Behavioral Control**				
I don’t feel comfortable going to a clinic to ask for birth control	60 (30)	23 (43)	19 (25)	18 (25)
The health care providers at the clinic would judge me if I asked for birth control	22 (11)	7 (13)	12 (16)	3 (4)
I don’t know where to get birth control	16 (8)	4 (8)	7 (9)	5 (7)

### Subjective norms

Subjective norms were among the most frequently identified barriers about contraception use. AYAs were frequently concerned about parental approval (n = 83, 42%) and that their friends might think they are looking for sex (n = 57, 29%; Table 4). Fifty-six (28%) reported concerns about convincing their male partner to use contraception.

### Perceived behavioral control

Few (n = 16, 8%) did not know where to get birth control but nearly one-third (n = 60, 30%) reported lack of comfort going to a clinic to ask for birth control and 22 (11%) reported concern about healthcare provider judgement when asking about birth control, more commonly reported among younger age categories (14–17 years, n = 7; 18–21 years, n = 12; 22–24 years, n = 3).

#### Qualitative findings

Our quantitative findings were similarly supported by our qualitative findings. Table 5 summarizes themes and provides illustrative quotes for qualitative findings related to each TPB construct regarding pregnancy and birth control use.

**Table 5:** Interview Themes and Quotes

TPB Construct and Interview Themes	Illustrative Quote
***Attitudes***: *Avoiding pregnancy is among biggest health concerns for AYAs*	I think people need to use birth control more, because we are living in a country where everything is impossible to afford. Insecurity is all over the country, and healthcare is non-existent. (23 years old) I am trying not to get pregnant right now, because I have already got a baby and I am still in high school. (19 years old) I am trying to avoid getting pregnant, because I am still in high school and jobless. In the situation that I am right now, I am surviving not living. (22 years old)
*AYAs commonly reported misinformation and concerns about contraception side effects*	I have heard using birth control can destroy all your white blood cells, it makes you cough up blood, weight loss. (14 years old) When I first started using birth control it made me have period(s) non-stop. It gives me short[ness] of breath. (19 years old) Some of them are scared to use it because they think it can make them develop some diseases. They think most of the nurses aren’t using birth control because they know it isn’t good. (19 years old)
*While very few had used a LARC, some noted potential benefits*	I like the fact I only have to go there once to have it inserted and don’t worry about going back until I want it to be removed. So, in a way I like the fact people don’t (know) my business, because people always think when you go there very often, it’s because you are looking for birth control. (23 years old)
***Subjective Norms*** *Most AYAs thought their partner’s opinion about contraception was important, however some were concerned about lack of partner’s support.*	We share the same opinions because he doesn’t want me to get pregnant. We think condoms are good to use. (18 years old) My actual boyfriend doesn’t want me to get pregnant right now because we are not ready. (23 years old) I don’t think he would have agreed for me to use it. (17 years old) I would want them to play a supportive role. Some agree, and some disagree for their girlfriends to use [it]. (20 years old) He had agreed for me to use birth control because he doesn’t want to get pregnant, but he doesn’t really like birth control in general. (25 years old)
*AYAs noted concerns about family and community judgement when seeking contraception.*	Somehow, I think my parents were the challenges because I couldn’t use any birth control, I wasn’t allowed. (20 years old) I think [young people are] afraid of what people might think when seeking help about birth control. (16 years old) [I have concerns about people that see you in PF (i.e., family planning) section, might go and tell your business in your neighborhood. (18 years old)
***Perceived Behavioral Control*** *AYAs commonly reported concerns about health care provider and community judgement.* *AYAs desired more information on contraception, preferably given in private from a doctor or nurse at a clinic that was not associated with maternal health care.*	I think they worried about what the nurses might say behind their backs. Also, about what people in the neighborhood might say. (18 years old) I would want a nurse. They usually make (an) educational class with a group of women; I think they need to set aside some privacy time in case I want to ask a question without having other women hearing to what I want to ask or say. (18 years old) I would want them to advise me how to prevent myself from getting pregnant again. And, how to keep myself in good shape, I would want all of this to be done privately. (19 years old) I would want them to tell me everything about birth control, their advantages, and disadvantages… in private. (24 years old)

### Attitudes

Most participants reported they would prefer to avoid pregnancy right now due to general insecurity they felt in their lives, as well as their desire to complete education and become more financially stable. Some describe barely being able to meet their general survival needs. For example, one participant stated “I think people need to use birth control more, because we are living in a country where everything is impossible to afford. Insecurity is all over the country, and healthcare is non-existent.” They also considered getting pregnant a difficult and costly experience, given the challenges of transportation and cost of prenatal and postpartum care. While most reported they could access free or low-cost contraception, most had limited sources of information regarding pregnancy prevention and contraception options. Misconceptions were common, as many AYAs reported misinformation about side effects and long-term health effects of contraception. For example, one participant stated “using birth control can destroy all your white blood cells, it makes you cough up blood, and weight loss.” AYAs reported they commonly share information about contraception between peers, however this frequently included sharing of misinformation about contraception in general and side effects. AYAs desired more access to contraception information, for example in response to the question “What would you want to know?” one participant stated “…all the valuable advice in private about how to use birth control and how to protect myself again sexually transmitted disease.”

### Subjective norms

Many AYAs reported they did not have conversations with their sexual partners about contraception or pregnancy prevention. Among those who did, they stated their partner’s opinion played an important role in their decision on whether to use contraception and what method they chose. While some noted their partners agreed with using contraception or condoms to prevent pregnancy, some also reported their partners did not agree or support their decision to use contraception. AYAs also harbored concerns about parental approval regarding contraception, as well as concern about community judgement, especially if they seek contraception care at a maternal health center. For example, in response to the question “In your experience, what are some challenges teens like yourself face in obtaining information about pregnancy prevention in general?” one participant stated “I think it is the lack of finding information and being afraid of what people might think when seeking help about birth control.”

### Perceived behavioral control

In interviews, AYAs generally felt like they knew where they could access free or low-cost birth control easily in their communities, but were held back by concerns about judgement from healthcare providers, particularly because they would have to seek contraception at a maternal health center where they would be visible to community members. Some also mentioned transportation challenges. AYAs commonly reported they would prefer to receive contraception education at either a generalized clinic or hospital rather than a school, in the community, or in a clinic associated with maternal health care. Education was preferred to be given by either a doctor or nurse. In addition, some stated the importance of this occurring in a private setting and via a female provider, for example one stated “I would want them to tell me everything about birth control, their advantages, and disadvantages… in private.”

## Discussion

In this multi-site cross-sectional mixed-methods study of AYA females living in Haiti, we found a large majority are sexually active and do not want to become pregnant. However, few reported use of effective contraception methods. While half reported contraception use at last sexual activity, most reported only condom use. Overall, AYAs reported using condoms less than half the time. Injectable contraception (i.e., medroxyprogesterone acetate) was the second most frequently reported contraception method, however only a small minority reported use of this method. Interview findings revealed numerous barriers to contraception access and use, including medication misconceptions and concerns about community, parental, and healthcare provider judgement. AYAs desired more information about contraception and would prefer to receive contraception education privately from a healthcare provider in a setting not affiliated with maternal health care.

Our study population reported higher rates of condom use and lower rates of injectable and LARC use compared with current literature on women of all ages in Haiti [3]. Past literature demonstrates that contraception and condom use differ by age in general, with those who are younger more commonly reporting condom use rather than other methods compared to older women [21, 22]. These differences are likely driven by numerous reasons, including healthcare factors (e.g., provider counseling, access) as well as psychosocial (e.g., stigma) and developmental factors (e.g., preference, ease of use) [23]. However, our study population reported lower rates of always/sometimes use of condoms compared to previous studies in Haitian youth [6, 8]. None of our participants reported use of LARC methods. As women living in rural Haiti have decreased access to all contraception methods compared to women in urban settings, [5] it is possible our findings are likely partially driven by contraception and condom access in the community rather than the participant’s preference. Also, while our partner organizations offer some SRH and contraception care in the two rural communities where enrollment occurred, we did not assess the specifics of the contraception care options offered at each site, and how this may impact our findings. Further study is needed to better understand how increasing access to condoms and all contraception methods, including LARC, impacts preferences and use in young women in rural Haiti, and how these may differ by age (e.g., younger age category (14–17 years) compared to older (18 years and older)).

We identified multiple barriers to contraception use. The most frequently cited barriers in our survey were concerns regarding side effects, parental approval, as well as peer or community judgement. This mirrors previous literature, especially for those living in LMICs [24, 25]. Some studies found that contraception programs that incorporate parental, peer and community engagement result in higher rates of contraception use among AYAs [26–28]. Perceptions about side effects were a noted concern, and many were rooted in misinformation about physical (e.g. loss of white blood cells) rather than psychosomatic side effects. As public health clinics in Haiti are required to provide contraception for free, [1] our study population did not feel that cost was a barrier and most stated they knew where they could obtain free or low-cost contraception. Studies investigating the impact of cost of contraception use in women in LMICs have shown conflicting results that to be driven by a number of factors [29]. Some studies have found those who are poorer and/or younger are more sensitive to contraception costs compared to less poor or older age groups [29]. However, AYAs reported concerns about SRH care access and transportation concerns, which are commonly cited barriers to contraception access for youth living in similar settings to Haiti [25, 30]. We also found that AYAs were concerned about privacy and may not attend clinics associated with SRH (i.e., maternal health or family planning clinic) for contraception due to concerns about judgement. Successful contraception programs for youth in similar settings have attempted to address this concern by offering contraception in non-traditional settings (e.g., schools, homes, satellite clinics) [24, 28]. Findings from interviews revealed that most would prefer to receive contraception education from a nurse or doctor at a clinic or hospital setting that is not affiliated or known for offering maternal health or contraception care. Thus, future efforts should focus on developing and testing innovative methods to offer contraception education and access for AYAs in Haiti in youth-friendly settings.

Our findings should be viewed in light of these limitations. We enrolled a convenience sample of AYAs thus there is risk for sampling bias. We enrolled relatively small sample size (n = 200); however we did enroll across two geographic regions to increase generalizability. In addition, due to the sensitivity of the topic, there is risk for potential inaccurate reporting. We attempted to mitigate this by engaging trusted community members and having young adult females collect data privately.

## Conclusion

Given the low rates of effective contraception use, inconsistent condom use, and high desire for pregnancy avoidance in our study population, future efforts are needed to increase contraception education and access for AYA females in Haiti. Using the TPB, we identified multiple actionable barriers and facilitators to contraception use among AYAs in Haiti. These findings can directly inform policy changes to support development of youth-focused interventions to improve contraception access in this population. Critical elements include engagement with parents, community members, as well as peers and sexual partners to increase support and acceptance of contraception programs for youth and ensure it is offered in an appropriate youth-friendly setting (i.e., not known for providing SRH care) and to empower autonomy in young women in rural Haiti. Further, it is vital to incorporate youth-specific training for HCPs to offer developmentally appropriate patient-centered contraception care. Given the numerous adverse health outcomes linked with unintended pregnancy in this vulnerable population, including high rates of maternal and infant mortality, this work has the potential to offset risk for these adverse outcomes in young women in Haiti.

## Electronic supplementary material

Below is the link to the electronic supplementary material.

Additional File: Interview Guide

## Data Availability

We do not plan to share individual participant data but study protocol, analysis plan, informed consent forms and clinical study report will be made available upon request by any researchers who provide methodologically sound proposal beginning 3 months and ending 5 years following article publication. Any researcher requiring more study information can contact the corresponding author (Masonbrink) of the manuscript to request sharing of study materials.

## Abbreviations

SRH: sexual and reproductive health.
LMICs: low and middle income countries.
STIs: sexually transmitted infections.
HIV: human immunodeficiency virus.
AYAs: adolescents and young adults.
TPB: theory of planned behavior.
HCP: healthcare provider.
LARC: long acting reversible contraception.
