# Supplementary material for: Sexual behaviors, contraception use and barriers among adolescents and young adults in rural Haiti
Source: BMC Womens Health. 2023 Mar 27;23:137. doi: 10.1186/s12905-023-02268-5 (PMC10045756; doi:10.1186/s12905-023-02268-5)
Supplement: Supplementary file 1 — Additional File: Interview Guide [file 12905_2023_2268_MOESM1_ESM.docx]

**Appendix: Interview Guide**

*[After informed consent]:* Thank you for agreeing to talk with us today. We are trying to develop health resources for young women in your community to support their sexual health and help them make informed decisions about pregnancy and birth control. Your thoughts and experiences about these issues will really help us.

I want to remind you that there are no right or wrong answers to the questions, we are really just interested in your own personal opinions. And remember, if there is ever a question you don’t feel comfortable answering, that’s perfectly fine. And as a reminder, there is no information attached to your answers that can be linked back to you (like your name). Do you have any other questions before we start? Do you mind if we start the audio-recorder?

*[Notes to interviewer: Bullet points are optional probes. Select as appropriate. Utilize general probes such as “tell me more” or “what do you mean by that” as needed to expand the discussion.]*

**How old are you?**

- When you are sick or need health care, where do you usually go? Tell me about the place.

*[optional probes:]*

- - How easy or difficult is it for you to get care?
  - How far is it it in relation to where you live?
  - How do you usually get there?
- Has there been any time when you thought you should get medical care, but you did not? Can you tell me about that experience?
  - When was this?
  - What made getting medical care too difficult to get?
- In the past year, have you received any sexual health care like testing for sexually transmitted infections and HIV?
  - If yes--Where did you receive this care? Can you tell me about that experience?
  - If no--If you wanted to get tested for HIV or other sexually transmitted infections, where would you go?
- In the past year, have you received any family planning counseling or services (like birth control), or prenatal health care?
  - If yes -- Where did you receive this care? Can you tell me about that experience?
  - If no --If you know where you could go if you wanted these services, where would you go?
- Thinking about young women around here are or that you know, how often do women think about their sexual health—this includes things like getting tested for sexually transmitted infections or using birth control?
- Thinking about young women in your community, what would you say are their biggest worries when it comes to their sexual health?
  - What do young women think about becoming pregnant?
    1. What if anything, concerns young women about becoming pregnant?
    2. Have you or anyone you have known had an experience where they became pregnant? Can you tell me a little about that?
  - What do young women think about using birth control?
    1. What are some reasons that women might think about or consider getting birth control?
    2. What, if anything, concerns young women about using birth control?
  - What do young women think about sexually transmitted infections?
    1. What, if anything, concerns young women about getting a sexually transmitted infection?

What are some reasons that women might think about or consider getting tested?

- The young women we have spoken to have a lot of different thoughts about when and if pregnancy is right for them. For you right now, would you say you are trying to become pregnant, trying to avoid becoming pregnant, or not sure? Why?
- What kinds of things in your life are important to you when making a decision about pregnancy?
- What are situations in your life that would make you feel you want to be pregnant?
- What are situations in your life that would make you feel you want to avoid getting pregnant?
- What do you think it would be like if you were to get pregnant right now?
- Have you been pregnant before? Could you tell me about that?
- Do you ever want to get pregnant [again]? When would be the ideal time?
- *[If trying to prevent]*: What were some of the biggest challenges you have faced when trying to avoid getting pregnant in trying preventing pregnancy?

*[If not trying to prevent]:*  Think about a time when you did not want to get pregnant. What was that like? What were some of the biggest challenges you faced in trying to prevent pregnancy?

- Can you share with me some of your general thoughts on birth control?
  - What have you heard about the different types of birth control?
  - Tell me about any experiences you’ve had with birth control- good or bad.
  - What makes using different birth control hard or inconvenient?
  - What role do men play in birth control?
  - What role would you like them to play in birth control?
- Are you currently using any birth control? What types?

If currently using:

- - What made you decide to try_____?
  - What do you like about____?
  - What do you not like about_______?
  - Some women we have talked to have had trouble using _____. Have you had trouble using___________?
  - What does your male partner(s) think about you using birth control?

If not using:

- - What are your reasons for NOT using birth control?
  - What do you like about NOT using birth control?
  - What do you not like about NOT using birth control?
  - What does your male partner(s) think about you NOT using birth control?
- Have you used any other types of birth control in the past?
  - What did you like about____?
  - What did you not like about_______?
  - What were the reasons you stopped using them?
  - Some women we have talked to have had trouble using _____. Have you had any trouble using__________?
  - What role did your male partner(s) play in using birth control?
- Have you ever been interested in using any (other) type of birth control?
  - What interested you about using________?
  - What do you think prevented you from trying________?
  - What else have you heard about_____?
  - In your opinion, what would be benefits of using ____ now?

In your opinion, what would be the downsides of using ____ now?

- *[If you have used birth control)* Where have you gotten your birth control, like what kind of place was it?
  - Tell me about the experience of getting birth control there.
  - What were the positive/negative aspects of your experience?
  - Tell me about the healthcare provider who prescribed you the birth control.
  - Tell me a little about the conversations you had with them.
  - What could have made your overall experience better?
- *[If not previously mentioned]*: There are some newer methods that are easy to use and long-acting, like an implant in your arm or an IUD (intra-uterine device). The implant is a matchstick-size, flexible rod that is put under the skin of the upper arm and takes just a few minutes to put it. The IUD is a small T-shaped device placed into the uterus by a doctor or nurse practitioner. Both methods work by stopping the egg from leaving the ovary, can stay in place for 3-5 years, are easy to take out, and are almost perfect at preventing pregnancy (99% effective).
  - What are your thoughts on these long-acting methods? What do you think of the IUD? What do you think of the implant?
  - Are these methods something young women who do not want to become pregnant might be interested in? Why or why not?
- Now, I’d like to understand more about romantic or sexual relationships in your life. Are you currently in a relationship with anyone or seeing anyone?
  - Can you tell me a little about this person? About how long have you two been together?
  - Is this a casual relationship or pretty serious?
  - Is this someone you have sex with or have had sex with in the past?
  - Do you have any other sexual partners right now? Can you tell me about them?
- Have you ever discussed pregnancy or birth control with your current main sexual partner(s) or partners in the past?

*[If yes, use questions below. If no, ask ‘what do you think your partner feels…….]*

- - What are their feelings about pregnancy?
  - What are their feelings about birth control?
  - What are their feelings about different types of birth control you mentioned?
  - If you were to get pregnant right now, how do you think your partner(s) would react?
  - How important are their feelings when you are making decisions about birth control?
- Before when we were talking about types of birth control, you [mentioned/didn’t mention] condoms. Can you tell me, the last time you had sex, did you and your partner use a condom?
  - Can you tell me a little about your reasons for [using/not using] condoms?
  - Have you used condoms before?
  - How does your partner/partners feel about condoms?
  - What discussions have you and your partner(s) had about condoms?
  - In what situations would you want to use a condom?
  - In what situations would you not want to use a condom?
  - Has there ever been a time that you personally wanted to use a condom but it didn’t end up working out? Can you tell me a little about that?
- Some of the young women I’ve talked with have told me about times that their sex partner or dating partner had physically hurt them by hitting, pushing, or choking them. How often does this type of thing happen to you or to the women you know?
  - If you were with a partner who did this, how would that affect your interest in getting on birth control or using birth control?
- Some young women I’ve talked with in the past have told me about times that their sex partner or dating partner had hurt them emotionally or tried to control them by saying mean things to them or reading their text messages. How often does this type of thing happen to you or to the women you know?
  - If you were with a partner who did this, how would that affect your interest in getting on birth control or using birth control?
- Are there any other person or people in your life whose opinions on pregnancy or birth control matter a lot to you?
  - Do you ever discuss birth control with them? What are those conversations like?
  - How do their views affect your decisions about birth control?
- In your experience, what are some challenges teens like yourself face in obtaining information about pregnancy prevention in general?
- What could help teens get information about how to prevent pregnancy or getting birth control?
  - What kinds of messages or information about sexual health and about pregnancy do you think is important to young women?
  - What do you think are the best settings, like at school, at a clinic or hospital, for teens to receive information or counseling about birth control?
- In your opinion, where would the best place for young women to talk to a doctor or nurse and actually get birth control if they wanted it? This could be any place—like a doctor’s office or somewhere in the community
  - Describe to me the ideal place right now for you to learn about and get birth control.
  - How do you think it would make you feel to be offered counseling and birth control at a clinic? Or at the hospital? Or at school?
  - Where do you think you would feel most comfortable discussing birth control with a doctor or nurse?
  - What would be the pros and cons (or good things and bad things) of going to the maternity center, Maison de Naissance?
    1. What are the problems or bad things that may prevent you from going to the maternity center, MN, to get birth control?
    2. What are the good things that may allow you to go to the maternity center, MN, to get birth control?
  - What would be the pros and cons (or good things and bad things) of getting birth control at hospital in Les Cayes?
    1. What are the problems or bad things that may prevent you from going to the local hospital to get birth control?
    2. What are the good things that may allow you to go to the local hospital to get birth control?
  - What would be the pros and cons of _______________ *(if they mentioned an alternative location during previous probes)*
  - Describe the ideal person you’d want to talk about birth control. Why?
    1. What would you want them to know?
    2. What would you want them to say?
    3. How should it be done?
- Thank you so much for sharing your thoughts with us. Is there anything else that we should have asked you about or anything else you’d like to tell us?
- Before we go, I just want to make sure I get a few things right about you and your experience. *[Review any demographic questions not answered clearly in the interview].*
- Do you have any questions for me before we go?
